# Standards-based curation of a decade-old digital repository dataset of molecular information

**DOI:** 10.1186/s13321-015-0093-3

**Published:** 2015-08-27

**Authors:** Matthew J Harvey, Nicholas J Mason, Andrew McLean, Peter Murray-Rust, Henry S Rzepa, James J P Stewart

**Affiliations:** High Performance Computing Service, Imperial College London, London, SW7 2AZ UK; Department of Chemistry, Imperial College London, South Kensington Campus, London, SW7 2AZ UK; Department of Chemistry, Centre for Molecular Informatics, Lensfield Road, Cambridge, CB2 1EW UK; Stewart Computational Chemistry, 15210 Paddington Circle, Colorado Springs, CO 80921 USA

**Keywords:** Digital repositories, Curation, Metadata standards

## Abstract

**Background:**

The desirable curation of 158,122 molecular geometries derived from the NCI set of reference molecules together with associated properties computed using the MOPAC semi-empirical quantum mechanical method and originally deposited in 2005 into the Cambridge DSpace repository as a data collection is reported.

**Results:**

The procedures involved in the curation included annotation of the original data using new MOPAC methods, updating the syntax of the CML documents used to express the data to ensure schema conformance and adding new metadata describing the entries together with a XML schema transformation to map the metadata schema to that used by the DataCite organisation. We have adopted a granularity model in which a DataCite persistent identifier (DOI) is created for each individual molecule to enable data discovery and data metrics at this level using DataCite tools.

**Conclusions:**

We recommend that the future research data management (RDM) of the scientific and chemical data components associated with journal articles (the “supporting information”) should be conducted in a manner that facilitates automatic periodic curation.

**Graphical abstract:**
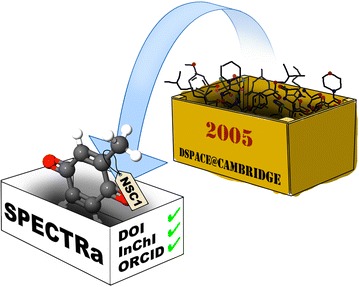
Standards and metadata-based curation of a decade-old digital repository dataset of molecular information.

## Background

Research data repositories based on platforms such as DSpace [1] were introduced about 10 years ago, and their use in domains such as chemistry and molecular sciences has gradually increased [2, 3]. Their importance has recently come to the fore with funding agencies in the USA, Europe and Asia all indicating that open deposition of research data will become a mandatory aspect of their funding, and many universities are now starting to consider the implications of implementing research data management, or RDM [4–6]. An early example of such RDM is illustrated with a project to produce a library of quantum-mechanically-optimised molecular coordinates derived from a computable subset of the National Cancer Institutes (NCI) collection of small molecules [7]. The information for each molecule was originally annotated by optimising the coordinates with respect to the energy obtained using the semi-empirical PM5 parameter set in MOPAC [8] (then the most current parameter set) and creating a DSpace collection. At the commencement of the present project, the original deposition of this information for 175,356 molecules into the institutional repository of the University of Cambridge [9] represented the only openly accessible copy.

An issue frequently raised in the context of research data management relates to the prospects of being able to access and use such digitally held information in the future. Relatively recently, such questions were largely directed towards the expected longevity of physical media such as punched cards and floppy disks (both now effectively extinct), hard drives, CDROMs, DVDs, magnetic tape etc. Few of these media have proven lifetimes exceeding 20 years and the real problem would be locating working devices capable of reading such physical media in the future. Quite different problems are associated with virtual collections, where the physical medium is less important than the information associated with the data itself. In this context, it is becoming increasingly accepted that successful long-term preservation of digital data depends upon repeated incremental improvements or curations taking place in 5–10 year cycles. Such operations can in principle be repeated indefinitely, thus creating a long-term mechanism with an anticipated lifetime of 100+ years if required. These curation cycles can track the evolution of data storage hardware, data formats and introduction of new software, so ensuring that the data remains accessible and in a usable form. The purpose of this project was to explore the viability of the long-term preservation of the 10 year old Cambridge dataset through such an incremental curation by performing its migration to the SPECTRa repository hosted at Imperial College London [2]. Specific benefits of undertaking such a curation include re-filtering the original source data for errors not previously eliminated, to produce an enhanced metadata record for each entry, and to recompute the optimised molecular coordinates by using the newer PM7 method. The original PM5 method used to obtain the molecular geometries was never formally published and is now unavailable, whereas the succeeding PM7 method has been formally peer reviewed and published [10].

We will also compare our approach with two other examples drawn from computational chemistry. The first [11] is typical of how almost all datasets derived from molecular computations are currently curated; in this case the stochastic generation of all possible stable molecular structures from an initial set of specified atoms. The trend in scientific publication in recent years has required authors reporting such studies to include more extensive data in the form of supporting information (SI) to accompany the scientific narrative from which their models are constructed and their conclusions drawn. We will argue here that these SI-based mechanisms for depositing, retrieving and re-using the data components of journal articles are no longer fit for this purpose (if indeed they ever were) and should be urgently replaced by repositories of data and closely-coupled metadata as a fundamentally different model for research data management. The second example describes [12] such a deposition of a dataset containing the quantum mechanically computed structures and properties of 134,000 molecules into the Figshare digital repository. We will ask here what the attributes of such a deposition must be in order to enable efficient formal re-curation 10 years after the original creation of the dataset, arguing that there are some essential structures and standards that must be fulfilled for such a process to be properly enabled.

## Methods

The migration of the original dataset was performed in three sequential phases, retrieval from the original repository, a technical validation and re-deposition into the SPECTRa repository.

### Retrieval

Both the Cambridge and Imperial-SPECTRa repositories are implemented using DSpace [1]. Although this software contains a component that can provide structured data representations of entries for harvesting (OAI-ORE [13] resource maps), this was not enabled on the Cambridge repository when we started our migration in July 2014. However, since the human-readable landing pages for each entry all conform to a structured HTML template, it nevertheless proved possible to extract all the data using ad hoc scripting and HTML processing (a process often informally referred to as “web-scraping”). This process was markedly inefficient, requiring three separate HTTP requests to the server per record, and took several days to complete. This approach is by no means unique; most large existing collections of (chemical) data require similar processes whereby a human has to initially read the documentation (if available) for the templates used to access the items and then to write appropriate custom codes or scripts to retrieve them. Such a method means that any unexpected change in the template resulting from, for example, the release of a new version of the dataset then inherits the risk of breaking these scripts. Stated more formally, the inferred uniform resource locators (URLs) for such collections of data are not persistent. The principal aim of our curation objectives therefore was to eliminate the need for such ad hoc scripting and replace it with a more efficient and standards-based workflow for achieving this persistence.

The following were retrieved from the original deposition [9] at Cambridge:

- The source URL for 175,356 records.
- 175,356 documents in XML-CML syntax encoded using chemical mark-up language (CML) [14], containing a molecular structure from the NCI database and some metadata describing the entry.
- 158,879 XML-CML documents containing the PM5 optimized coordinates of the NCI database structure and basic metadata, including the NCI identifier for version 3 of the NCI Open Database and the computed InChI and InChIkey [15]. Of these, 158,122 were found to be unique. The remaining 16,477 entries had no reported PM5 calculation. These entries were previously identified [7] as having additional complexities such as the presence of metal atoms or problems with correctly adding hydrogen atoms and charges, and so a PM5 calculation had not been attempted. Here we have adopted the same strategy of not recovering these entries in the present curation.

### Technical validation

No metadata were provided in the original depositions that gave an unambiguous description of the two XML-CML documents present in the form of a CML schema declaration, and no MOPAC version information or MOPAC input or output files were saved to act as alternative sources of this information. The CML syntax corresponding to the annotation derived from PM5 optimisation in the form of files named e.g. nsc138467_post-mopac.cml in the original collection was incomplete; bond connection terms were missing and the CML documents failed validation according to the CML Schema version 2.4 [16]. The first task was therefore to develop a protocol to produce a reliable and valid input file suitable for re-calculating the properties using the newer PM7 method [10]. Many entries in the NCI collection comprise two or more disconnected components, of which only the larger component was retained in the original editing [7]. The resulting missing component in the starting structure was predominantly a counter ion and its removal requires a charge to be assigned to the remaining fragment. This information was originally captured in both original XML-CML documents, the first as part of an identifier element containing an early form of the InChI string:

$$\tt{<\!identifier\, version="0.932Beta" \,tautomeric="0"> \:<\!basic\!>C13H21N2O,\,1H3\text{-}12H(2H3)15(13H(3H3)4H3)11(16)10\text{-}7H\text{-}6H\text{-}8H\text{-}14(5H3)9H\text{-}10<\!/basic\!> <\!charge\!>+1<\!/charge\!> <\!/identifier\!>}$$

The second is declared more formally in the CML molecule element associated with the PM5 calculation:

$$\tt{<\!molecule \,id="NSC138467"\, formalCharge="1"\, name="mol1"\!>\,}$$

Of the 158,122 unique documents in the latter category, the formalCharge declarations were distributed as follows; 153,127 (0), 28 (−1), 4,456 (+1), 18 (−2), 483 (+2), 2 (−3), 3 (+3), 1 (+4), 4 (−5). Manual inspection of the species with very large formal charges (>|3|) indicates these are all errors arising from the original curation process because of incorrect interpretations of e.g. metal centres. Our original attempt to transform this information into a MOPAC input involved the standard OpenBabel [17] program, version 2.3.2. It transpired OpenBabel did not correctly propagate the charge information in either of the original CML files by transformation into an appropriate MOPAC keyword declaration such as CHARGE = 1. Instead the generic statement $\tt{PUT\, KEYWORDS\, HERE}$ was the only content of the MOPAC keyword line. This raises some interesting issues:

1. Absolute fidelity in any syntactic transformation of data from one format to another is very difficult to achieve. Thus there are often multiple syntaxes for any given information field, such as the two shown above for expressing the charge on a molecule, and all such variations must be honoured with complete fidelity to achieve reliability. Although some forms can be quickly deprecated (such as the first example above), these forms cannot be ignored and they must be processed.
2. The MOPAC program does not mandate the presence of all keywords. A calculation may still succeed on the assumption that a missing keyword simply defaults to a pre-determined value. In this case, MOPAC will assume that the value of an undeclared CHARGE keyword corresponds to zero, which is a clear error if the charge was intended to be non-zero. This issue of *implicit semantics* is perhaps the single largest problem in ensuring validation. It can be very difficult, if not impossible to find complete definitions of what implicit assumptions are made in any system. Often the only source of these is the actual computer code itself.
3. A further implicit rule for MOPAC keywords is that the spin-multiplicity of the system is computed from the total electron count after the appropriate charge is applied. For a system where a charge of e.g. +1 is left undeclared, that will result in a molecule with an odd number of electrons, and this is then treated implicitly as a molecule with a $\tt{DOUBLET}$ spin state. We also note that these implicit rules are not universal; other programs such as Gaussian use different conventions.
4. If the explicit keyword $\tt{SINGLET}$ (spin state) is declared, a safe assumption for virtually all real molecules that exist as physical samples, this can act as a checksum. The MOPAC program will then throw an error and the calculation will not proceed if this spin state conflicts with any declared or undeclared/implicit charge.

Instead of using OpenBabel, we made a custom conversion of the original post-MOPAC PM5 calculation into CML files to ensure the correct keywords were written to the MOPAC input file. The atom positions were expressed in internal coordinates rather than cartesian coordinates. This is not a critical decision, since the final atom positions do not in general depend on the initial coordinate system selected. A PM7 geometry optimisation was then performed using the resources of the Imperial College High Performance Computing Service. The majority of calculations completed within tens of seconds and the total required approximately 20 CPU days of computer time.

#### InChI identifiers

An InChI identifier [15] is a canonicalization based on the atom connectivity of a molecule, which in turn is derived from Cartesian coordinates for each atom using simple heuristic rules specifying a range of atom pair distances for any element combination. These distance ranges are built into OpenBabel [17]. Unfortunately, atom connection distances are not formally defined as accepted standards, and the precise values are ultimately the choice of the designers of any program implementing them. The limits however are usually sufficient tolerant to cover the vast majority of real molecules without any disagreement, and this would especially be true of the NCI set which cover real systems rather than hypothetical or computed molecules. This does not entirely exclude there being a very small number of molecules where specific atom-pair distances might fall within e.g. a bond range using PM5-optimised coordinates but which are e.g. outside such a range using PM7 values. We note that whilst it is possible to replace these relatively arbitrary rules by using a quantum mechanically derived property of the electron density topology called the BCP (bond critical point) to define an atom-pair connectivity [18], this is not currently used for determining InChI identifiers.

We proceeded to derive InChI identifiers using the following OpenBabel [17] commands:

1. $\tt{babel \text{-}i \,cml\, nsc383508\_original.cml\, \text{-}o\, xyz\, out.xyz\, \text{-}\text{-}canonical}$ (for the original NCI-based data)
2. $\tt{babel \text{-}i\, cml \,nsc383508\_post\text{-}mopac.cml\, \text{-}o\, xyz\, out.xyz\, \text{-}\text{-}canonical}$ (for the PM5-computed data [7])
3. $\tt{babel \text{-}i\, mopout\, MOPAC\text{-}PM7.out\, \text{-}o \,xyz\, out.xyz\, \text{-}\text{-}canonical}$ (for the newly generated PM7-computed data).
4. $$\tt{babel \text{-}i\, xyz\, in.xyz\, \text{-}o\, inchi\, out.inchi}$$

Commands 1–3 convert all the data into Cartesian coordinates to remove any possible atom connection data that might have been generated by MOPAC or other sources. Command 4 generates a canonical InChI identifier [15] using these coordinates. This process ensures that the connectivities created using the last command and then used to create the InChI are normalised against a single connection algorithm (being the one contained in OpenBabel, version 2.3.2). These InChI strings are then compared with those derived in a similar manner using the original NCI and the original PM5 computed coordinates (Table 1).

**Table 1:** Comparison of generated InChI identifiers

NCI = PM5 = PM7	(PM5 = PM7) ≠ NCI	(PM7 = NCI) ≠ PM5	(PM5 = NCI) ≠ PM7	PM7 ≠ PM5 ≠ NCI
154,552	2,041	573	470	486

Of the 158,122 unique values (Table 1), 97.7 % matched for all three instances, which provides a great measure of confidence that the atom-connection algorithm is robust. To identify the origin of the 2.3 % of InChI mis-matches, we have to dissect the InChI identifier itself into its component layers:

1. The molecular formula layer (1131).
2. The pairwise atom connectivity layer, determined as described above (127).
3. The hydrogen layer, in which hydrogen atoms are added to all heavy atoms where a valence is perceived to be unsatisfied if the hydrogens are not already declared. Because we have subjected all the systems to computational quantum modelling, all hydrogen atoms are already explicitly defined in our coordinates (1252).
4. A charge layer, also defined for all the molecules in our collection (9).
5. A stereochemical layer. Because our coordinates are all specified in 3D space, the stereochemistry is always defined. This layer includes double-bond isomerism (292) and tetrahedral configurations (267).
6. An isotope layer (22).

The distribution of the 2,997 differences between the PM7 and the NCI InChI identifiers (2,041 + 470 + 486, Table 1) are shown in parenthesis in the listing above, and each is very briefly discussed below:

1. The discrepancies in the formula layer originate from mismatches in the hydrogen count. This is because, historically, molecules were not always defined with explicit coordinates for all hydrogen atoms. Instead they were inferred from residual valences, these in turn inferred from bonding angles and other geometric and heuristic information. The process of replacing such implicit hydrogens with explicit ones is not always exact.
2. The connection layer mismatch originates from bonds that are on the verge of connection and derives from (possibly small) geometric changes from the quantum mechanical re-optimisation. A typical example of such uncertainty are putative S…S bonds in sulfur species [19].
3. This and the formula layer together account for the great majority of the mis-matches.
4. The small number of mis-matches in the charge layer may result from the InChI code heuristic for deciding the appropriate charge for a molecule. As noted above, we detected some unreasonable high charges resulting from this process.
5. Because traditionally molecules were expressed in the MolFile V2 format which allows just 2D coordinates to be defined, stereochemistry had to be added using an additional parameter associated with each bond connection and equivalent to the stereochemical wedge notations used in organic chemistry. This information is not free of ambiguity, since the stereochemistry is defined relative to other atoms and can lead to logical contradictions. When such two dimensional coordinates and this additional information is converted into 3D coordinates (a process carried out during the original deposition [7]), ambiguities can result.
6. Isotopes were not included in the MOPAC-PM7 calculation.

### Re-deposition

For each remaining entry, the PM7-derived InChI strings and keys were added to the SMILES strings and the NCI and CAS accession identifiers obtained from the original data and propagated as metadata. We note that the NCI identifiers themselves may not necessarily persist across different versions of the NCI database, which was version 3 at the time of the original curation and has subsequently been updated to version 4 in 2012 [20].

Prior to import to SPECTRa, each entry was packaged individually to produce an archive file, termed a *SWORD* [21, 22] bundle. SWORD (Simple Web-service Offering Repository Deposit) is an interoperability standard for data ingest into digital repositories, rendering these bundles suitable for import into any SWORD-compliant repository, not just Dspace-based SPECTRa. The bundles contains a METS manifest [23] and data files and were created using a locally written tool.

The METS manifest contained the following metadata:

- InChI and InChIKEY and SMILES string.
- CAS and NCI accession IDs, NCI entry name.
- Back-link back to the entry in the Cambridge repository.
- DOI link to the published description [7].
- ORCID [24] identifiers for the contributing authors.
- Link to Creative Commons License terms.

The datafiles included within the bundle were:

- Two CML files [14] containing unaltered copies of the NCI coordinates [20] and PM5 computed MOPAC output documents obtained from the original source repository.
- A third CML file conflating the three previous structures, containing the original NCI structure, the original PM5 structure from the original repository and the newly computed PM7 structure.
- MOPAC input and output files for the new PM7 calculation.

Import of this fileset to the destination SPECTRa repository was performed using the SWORD web service interface. Owing to a limitation of the Dspace-SPECTRa SWORD interface, no bulk-import function was available and all of the new packages had to be to uploaded individually, a process that took approximately 60 days. Doubtless this exceptionally long time resulted from some undiagnosed server misconfiguration and should not be considered a representative characteristic.

### Exposing the metadata structures on DSpace-SPECTRa

**Fig. 1:**
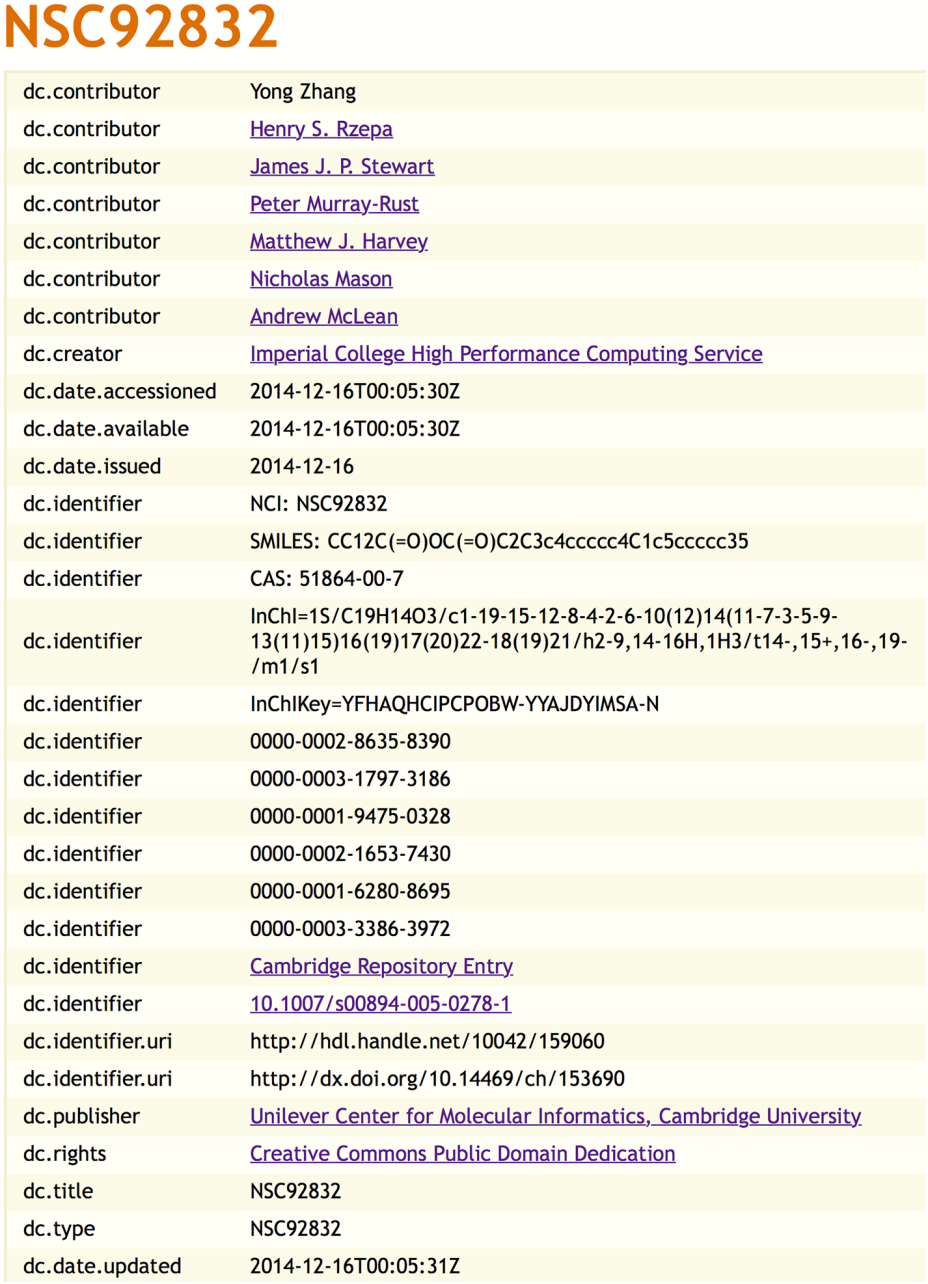
Metadata information associated with the curated dataset.

**Fig. 2:**
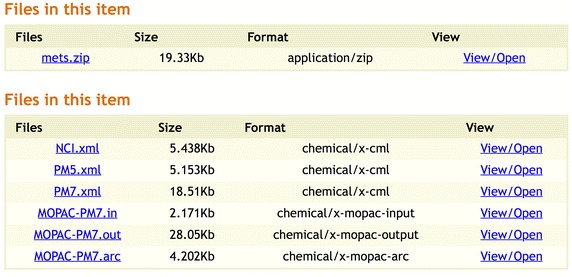
File bundles associated with the curated dataset [26, 27].

The outcome of the curation process resides in a new collection on the SPECTRa repository comprising 158,122 entries. The new curation has two persistent identifiers for the collection itself [25] and within that collection, individual molecular entries are themselves also assigned two persistent identifiers, as for example the entry shown in Figs. 1 and 2 [26, 27]. The first of these is the handle with a registered prefix *10042* associated with the SPECTRa DSpace server. The second is the DataCite DOI associated with the prefix *10.11469* as registered to Imperial College, with individual entries prefixed with the common string *ch***/** to indicate the chemistry department at that institution. The individual items in the collection also have a full set of associated metadata descriptors (Fig. 1).

Newly introduced metadata since the creation of the original collection include the following:

- The contributors are listed individually, with each name linked to their corresponding ORCID [24] entry page.
- The computational resource used for annotation is also linked with a non-persistent identifier; currently to the Web landing page for the organisation.
- Several chemical identifiers are included such as SMILES, InChI and the CAS accession number. The significance of including such metadata is that it is registered automatically with DataCite.org [28], and hence available for fielded searches [29].
- The ORCID entries [24] for all collaborators are explicitly listed, and again become available for searching [29].
- A back-link to the original item deposition [9] allows comparison of the original and the newly curated entry. Because this handle prefix (1810) is unregistered with CNRI, the central authority for Handle registration, it cannot be treated formally for resolution as a persistent identifier. This is one of the aspects we wished to rectify in the current curation.
- There is also a persistent identifier link to the journal article [7] describing the original work. In due time, the present article could itself be so-referenced in a future curation.
- A pair of new persistent identifiers for each molecule has been minted as part of the curation. The first is a handle assigned using the Handle manager tool in DSpace itself and which can be resolved using either of the services http://hdl.handle.net/ or http://doi.org/. This handle is internally annotated with 10320/loc records [30] to enable automated retrieval of individually requested files from the deposition. The prefix *10042* is registered to the SPECTRa server.
- The second persistent identifier [27] is assigned using the DataCite API [28], and serves as a mechanism to allow DataCite to acquire the metadata for this entry. The prefix *10.14469* and the suffix *ch* are as described above.
- A (non-persistent) link to the original publisher is included.
- A (non-persistent) link to the open license for the data, in this instance Creative Commons Attribution (CC0) [31]. It is perhaps surprising that this license is itself not identified by its own persistent identifier, but the URIs for the CC licenses and the corresponding resources are however machine-processable.

This metadata describes the contents of the data files resident for each entry (Fig. 2).

The fileset for the deposition comprises two so-called bundles. The first item is identified internally as the SWORD bundle. This compressed archive contains the METS manifest [23] for the deposition, expressed syntactically as an XML file containing a number of declared namespaces defining various metadata schemas. The METS manifest, along with another internal XML document, the OAI-ORE resource map [13] defines the contents, locations and properties of the documents comprising the collection.

The second item (Fig. 2) includes three XML files expressed syntactically as XML documents declaring the CML schema [16]. One can find a semantically rich encoding of the molecular information within each file. Also included in this fileset are three files relating to the MOPAC program: the input file, the corresponding PM7 output and PM7 archive file summarising the computed properties. In principle, all the information in these files could also be absorbed into the CML descriptors, although this has not been done in the present instance. These files in turn have associated MIME types [32], information that allows automated retrieval of the files using one of the mechanisms briefly described below.

### Metadata interfaces to DataCite

In curating the original collection at Cambridge by relocating it to a separate DSpace server, we wished to ensure that new persistent identifiers for each entry could be minted using DataCite. That in turn required the metadata follows the Dublin Core Schema held on the DSpace-SPECTRa repository to be mapped onto the DataCite Schema using an XSLT-based crosswalk transform. The following procedure was used to achieve this.

- A recent release of DSpace (DSpace4) largely automates the minting of DOIs using DataCite. Our target DSpace (SPECTRa) is running version 1.8; the DOI module for DSpace 4 is confined to a few distinct packages that were implemented into version 1.8 without affecting the other components. The following Java packages were extracted from DSpace4 and used within DSpace 1.8:
  $$\tt{org.dspace.identifier.doi,\, org.dspace.services,\, org.dspace.versioning,\, org.apache, \,httpcomponents,\, httpclient\text{-}4.2.jar,\, org.apache.httpcomponents.httpcore\text{-}4.3.1.jar}$$
- DOI-specific properties in the existing install were configured via dspace.cfg. An auxiliary configuration file spring-dspace-addon-identifier-services.xml is packaged within the org.dspace.identifier package and used for connection details.
- Configuring the XML schema transformation that translates or “crosswalks” between the DSpace Dublin Core metadata schema and the DataCite metadata schema. DSpace4 delivered the requisite crosswalk, DIM2Datacite.xsl, for version 2 of the DataCite schema.
- A requirement was to provide metadata that described the locations, filenames and file types of the individual datafiles associated with each DOI, in order to provide a machine discoverable and operable path from the DOI directly to the files containing chemical data. To achieve this, the DSpace 4 XML schema transformation (crosswalk) was extended to include the locations of the METS and OAI-ORE metadata files that are generated by DSpace, as relatedIdentifiers. These related identifiers used the HasMetadata relation type which was introduced in version 3.0 of the DataCite Schema:

$$\tt{<\!relatedIdentifier\, relatedIdentifierType="URL" \,relationType="HasMetadata" \,relatedMetadataScheme="METS" \,schemeURI="http{:}//www.loc.gov/METS/"\!>}$$

$$\tt{https{:}//spectradspace.lib.imperial.ac.uk{:}8443/metadata/handle/10042/159060/mets.xml}$$

$$\tt{<\!/relatedIdentifier\!>}$$

Both the METS and OAI-ORE files contain the desired metadata and can be processed as required. As an example, the $\tt{fileSec}$ section of the METS is show in part below:

$$\tt{<\!mets{:}file CHECKSUMTYPE="MD5" GROUPID="group\_file\_1367638"}$$

$$\tt{ID="file\_1367638" \,MIMETYPE="chemical/x\text{-}cml" \, SIZE="18955"\, CHECKSUM="88761c87f8f090182d910f33a7467435"\!>}$$

$$\tt{<\!mets{:}FLocat\, LOCTYPE="URL"\, xlink{:}title="PM7.xml"}$$

$$\tt{xlink{:}type="locator"}$$

$$\tt{xlink{:}href="/bitstream/handle/10042/159060/PM7.xml?sequence=3"/\!>}$$$$\tt{<\!/mets{:}file\!>}$$

- The crosswalk was also extended to add metadata for ORCID as name identifiers for the contributors and various chemical identifiers (InChI, InChIKey, CAS, NCI and SMILES) as a set of alternate identifiers.
- In addition, the PM7.xml file containing the newly computed structures and properties was registered against its chemical MIME type [32], *chemical/x-cml* for each DOI, using the DataCite Media API. The DataCite content resolver [28] then allows this CML file to be directly retrieved from the associated DOI through content negotiation using the resource http://www.crosscite.org/cn/ or directly by URL.

It took around 8 h to mint DOIs for an initial run of 23,240 items, and further subsequent updates took only a few hours. Each update required about 24 h to become visible in DataCite. New items in the repository are now synchronised hourly using the DSpace4 programme, RegisterDOI.

At this stage, the DataCite Search API [29] proved to be a useful tool for checking the quality and validity of the curation and its metadata. Search queries were used to retrieve lists of all entries belonging to the new DSpace-SPECTRa collection in an easily parsed format and with the necessary metadata to identify discrepancies, such as duplicate DSpace depositions, duplicate assigned DataCite DOIs or corrupted or invalid metadata. Some examples of such use are collected in Table 2 and are also described below. An advantage is that this kind of analysis can be done without privileged access to the host repository and its underlying databases, which makes it easier for peers and users to scrutinize the quality of large open data collections and flag any potential errors.

**Table 2:** Examples of data discovery and datametrics using metadata

Entry	URL	Purpose of search
1	http://search.datacite.org/api?&q=prefix:10.14469&alternateIdentifier:NCI\:*&fl=doi,title,relatedIdentifier&wt=xml&rows=3	Returns the first three entries for which any NCI descriptor is specified, restricted to the Imperial College prefix, containing values for the title, doi and RelatedIdentifier and expressed in XML syntax
2	http://search.datacite.org/ui?&q=alternateIdentifier:smiles\:*.*+alternateIdentifier:NCI\:*	This returns all entries for which both a SMILES and NCI molecular descriptor is specified and which contains a period in the SMILES string
3	http://search.datacite.org/ui?q=ORCID:0000-0002-8635-8390+publicationYear:[2014+TO+2014]	This returns the metadata about the ORCID associated with the creator of a data set, along with a specified period for its creation
4	http://search.datacite.org/ui?q=ORCID:*+prefix:10.14469	A variation of the preceding example, illustrating all entries at Imperial College that have an associated ORCID for their creator
5	http://search.datacite.org/ui?q=ORCID:*+doi:10.14469\/CH\/*	A second variation of the preceding example, illustrating all entries at Imperial College that have an associated ORCID for their creator and a DOI assigned to the Chemistry department
6	http://search.datacite.org/ui?q=has_media:true&fq=prefix:10.14469	Searches for any entry associated with a declared media type. The prefix is that registered by Imperial College London; the media type found for this prefix is chemical/x-cml
7	http://search.datacite.org/ui?q=InChIKey=LQPOSWKBQVCBKS-PGMHMLKASA-N	A search using a specified value for the InChI chemical identifier associated with the dataset. Our repository was constructed along the lines that each deposition describes a single molecular collection, where a single InChI descriptor relates to the important molecular entity in that collection
8	http://search.datacite.org/ui?q=alternateIdentifier:InChIKey\:*	A variation on the preceding specific search, where all entries containing an InChIKey are returned
9	http://stats.datacite.org/?fq=datacentre_facet%3A%22BL.IMPERIAL+-+Imperial+College+London%22&fq=allocator_facet%3A%22BL+-+The+British+Library%22&q=#tab-resolution-report	This provides a URL resolution report for all DOIs associated with the Imperial College London prefix
10	http://stats.datacite.org/?fq=datacentre_facet%3A%22BL.IMPERIAL+-+Imperial+College+London%22&q=#tab-prefixes	This returns the number of datasets associated with Imperial College as a whole

## Results and discussion

The configured metadata infrastructures now associated with each item in the collection enable individual datafiles to be accessed based only on knowledge of the persistent identifiers and media type, which can be allowed to default to specific type. We have implemented three procedures for doing this; these are fully described elsewhere with discussion of the pros and cons of each approach [33, 34]. Here we provide only a summary of these methods.

1. The first access method to be developed [33] is based on extensions to CNRI Handle record types known as 10320/loc [30]. These allow the handle record to be retrieved using the Handle REST API, which allows programmatic access to handle resolution using HTTP. A typical invocation would be using a URL of the type http://doi.org/10042/31117?locatt=mimetype:chemical/x-cml where the string 10042/31117 is the assigned Handle identifier and chemical/x-cml the requested media type.
2. The DataCite Media API also allows a DOI to be resolved based on the media type of the required document, typically a URL of form http://data.datacite.org/chemical/x-cml/10.14469/ch/153690, where the string 10.14469/ch/153690 is the assigned DataCite identifier, and chemical/x-cml the requested media type [34]. This URL can be passed to any requesting program and the file associated with this information will then be retrieved from the repository.
3. OAI-ORE Resource Maps exposed through DataCite metadata. We have made the OAI-ORE Resource Map [13] and the METS manifest [23] (both generated internally by Dspace) discoverable by including their locations as relatedIdentifiers within the DataCite metadata for the dataset [35], as described above. This allows a script to query for example the resource map to retrieve the URL associated with the data file. Again, the only information required by the script is datacite_jmol(‘10.14469/ch/153690?chemical/x-cml’), where datacite_jmol is the Javascript function written to process the responses [36].

Any of the above methods [34] can be used in conjunction with e.g. a visualisation program which can convert the data contained in the retrieved file into a graphical representation, or as part of a script which could retrieve a greater number of files for the purpose of e.g. data mining.

## Data discovery and datametrics

Enhancement of the original Cambridge dataset with the features described above greatly improves the discoverability of the data. Enriching metadata and then exposing it in a manner that allows the Datacite organisation to harvest it enables exploitation using the DataCite interface [29] and allows statistics to be collected [37]. Examples of both are shown in Table 2.

The current DataCite search resource is still styled *beta*, and it is probable that the features offered in the future will become greatly enhanced.

## The benefits of achieving SWORD/OAI-ORE and METS-enabled endpoints

Perhaps the most significant technical improvement realised as a result of this activity is the facilitation of future curation efforts, as part of a strategy to address the issue of what has graphically been described as *link rot* [38], whereby a worryingly large proportion of non-persistent identifiers used to cite data and associated information are found not to link correctly after just a few years or in some cases months. Digital repositories are intrinsically designed to enable replication of content to other locations whilst preserving essential information such as persistent identifiers. Here we focus on the DSpace repository, which provides an *OAI-ORE* endpoint implementing the Open Archives Initiative’s Object Reuse and Exchange standards [13] to achieve such replication. The ORE manifest for the deposition illustrated in Figs. 1 or 2 for example is declared in metadata as: $\tt{https{:}//spectradspace.lib.imperial.ac.uk{:}8443/metadata/handle/10042/159060/ore.xml}$ or $\tt{https{:}//spectradspace.lib.imperial.ac.uk{:}8443/metadata/handle/10042/159060/mets.xml}$ for the METS manifest (see above). These locators derive from the assigned handle for this entry as 10042/159060. For each entry, a structured XML representation of the data (for example PM7.xml), including a declared standard XML schema (CML 2.4) is included. This allows the data to be directly parsed using a generic XML import/export tool, so enhancing any future wholesale export of the dataset. The use of XML is to be preferred to older legacy chemical formats, for which no explicit schemas are, or indeed can be, declared.

The following illustrates a programmatic method for a curation procedure that could be employed if starting from a SWORD [21, 22] /OAI-ORE [13] and/or METS-enabled endpoints.

- Obtain a list of all the individual entries for the collection. This is accomplished by using DataCite to search for any unique identifier associated with the collection, which is defined in this example by the string alternateIdentifier:NCI:
- This can be accomplished using the command: $\tt{curl}$ http://search.datacite.org/api?&q=prefix:10.14469&alternateIdentifier:NCI\:*&fl=doi,title,relatedIdentifier&wt=csv&rows=170000-oNCI.csv.
  The value 170,000 in this string is the expected upper bound. The prefix *10.14469* restricts the search to collections at Imperial College only (to disambiguate from any other collections with the same name elsewhere).
- This returns the following information for each entry (in this example in csv format, with other options being XML, OAI-PMH or json): $\tt{doi,title,relatedIdentifier 10.14469/CH/123315,NSC5396, "HasMetadata{:}URL{:}https{:}//spectradspace.lib.imperial.ac.uk{:}8443/metadata/handle/10042/130536/mets.xml, HasMetadata{:}URL{:}https{:}//spectradspace.lib.imperial.ac.uk{:}8443/metadata/handle/10042/130536/ore.xml,\, IsPartOf{:}Handle{:}10042/31117"}$

This reveals that ORE and METS manifests are associated with the *Related identifier* metadata element, and the direct path to each is obtained from the value of the *HasMetadata* child.

- These provide programmatic access (using XSLT transforms or other methods) to the METS bitstream itself, which contains all the files in the deposition as a compressed archive. The METS bitstream has the URL: $\tt{https{:}//spectradspace.lib.imperial.ac.uk{:}8443/dspace/bitstream/handle/10042/31117/mets.zip? sequence=8}$
- This is retrievable using:$\tt{curl\, https{:}//spectradspace.lib.imperial.ac.uk{:}8443/dspace/bitstream/handle/10042/31117/mets.zip?sequence=8}$.
- Each METS manifest can then be injected into the destination repository, with the string *10042/31118* defining the Handle for the entire new collection (not that for the individual entries):

$$\tt{curl \,\text{-}si\, \text{-}\text{-}data\text{-}binary @mets.zip\, \backslash}$$$$\tt{\text{-}H\, "Content\text{-}Disposition{:}filename=mets.zip"\, \backslash}$$

$$\tt{\text{-}H\, "Content\text{-}Type{:}application/zip"\, \backslash}$$

$$\tt{\text{-}H\, "X\text{-}Packaging{:}http{:}//purl.org/net/sword\text{-}types/METSDSpaceSIP"\, \backslash}$$

$$\tt{\text{-}H \,"X\text{-}No\text{-}Op{:}false"\, \text{-}H "X\text{-}Verbose{:}true"\, "https{:}//USER{:}PASSWORD@spectradspace.lib.imperial.ac.uk{:}8443/sword/deposit/10042/31118"}$$

Finally in this section we note PREMIS, another international standard for metadata supporting the preservation of digital objects to help ensure their long-term usability [39]. Currently, the PREMIS Schema is only used in DSpace instances to represent technical metadata about DSpace bitstreams (i.e. files), being generated by a PREMIS crosswalk.

## Comparison with other repositories

Here we compare our approach for data deposition with that of two alternative existing data repositories, one of which is also based on DSpace (Dryad [40]) and a second Figshare [41] that is not. The first is run as a not-for-profit organisation that offers data deposition services, with persistent identifiers provided by both the DSpace handle manager and also via DataCite. Dryad deploys a subset of the metadata configured for our SPECTRa server, but significantly this does include [42] an OAI-PMH based programmatic method for access to the data object via the METS manifest, allowing a procedure similar to the OAI-ORE resource map outlined above to be used to access the datafile. Dryad differs in one significant regard from our approach in terms of the granularity of the deposition. Since our data is based on the computed properties of discrete molecules, we have adopted the strategy of one data record per molecule, and hence the dataset for each molecule is also assigned its own persistent identifiers. In contrast, the primary model used by Dryad offers coarser granularity of one data record per associated publication whereby the complete Dryad data set is linked with a peer reviewed journal publication. The net result is a pair of persistent identifiers, one for the article and one for the data, with the data component embargoed until the article itself is released after peer-review into the public. We do not regard this approach as an optimal one when dealing with molecular data, since it cannot permit any discovery process for individual molecules contained in such a collection.

DOIs can also be minted using the current (2015) version of Figshare using the DataCite API. This commercial repository is not currently OAI-PMH/OAI-ORE compliant and so no standard ORE or METS resource maps are declared to DataCite using e.g. the *related identifier* element of the DataCite metadata schema. This lack of OAI-PMH/OAI-ORE compliance would render a lossless curation of our SPECTRa collection to e.g. Figshare more difficult to achieve programmatically, but such an operation is not excluded in the future when the functionality becomes available.

## Comparison with two other collections of molecular quantum mechanical calculation data

We first return to discussing the article reporting the results of a stochastic exploration of the structures predicted using quantum mechanical procedures [11]. Initial approximations based on approximate methods are refined using much higher levels of theory. The molecular coordinates for unexpected, unusual or interesting outcomes from this procedure were then deposited into the supporting information (SI) associated with the published article. This contains just 10 species, although clearly far more molecules were computed at various levels of theory and these now appear lost to science. The SI itself takes the form of a paginated PDF file downloadable from the article landing page, and which contains no exposed associated metadata for any individual entry. Discussion is included here because it is very typical of the data associated with studies of this type. Curation of such data is really only worthwhile if it is first aggregated into a larger collection, a process that is never attempted because of this formal lack of metadata. The resulting fragmentation and hence loss of valuable data is, we argue, one of the broken aspects of current publishing models that require urgent attention.

As with the previous example, the next article [12] describes quantum mechanics based procedures to obtain the molecular structures of a much larger collection of 134 kilo-molecules and the subsequent methods involved in creating a digital repository based collection of these. Depositing all the calculations recovered from this process goes one important stage beyond the previous example, and is therefore to be welcomed. However, an important unanswered question is how easy would it be to curate this collection in a decade from now. In fact, several fundamental design features [12] have made such an operation unnecessarily difficult.

- The entire dataset is associated with a single persistent identifier [43] expressed as a compressed archive that a user can download and expand into a folder containing 133,886 individual files. The collected metadata however does not refer to these files, but to the folder containing them, which in turn means that the contents of this folder are in effect not discoverable using the mechanisms described above.
- In general, it is quite difficult on most computer systems to navigate a single folder containing 133,886 items. One would have to resort to using specialised software to do this, and this would probably restrict inspection to individual files and not to a sub-collection with specified properties.
- The individual entries adopt the original XMol XYZ syntax. That syntax has then been annotated with a number of other properties, both to the individual atoms and to the molecule as a whole, the latter including both SMILES and InChI strings. Unfortunately, this annotation is in effect ad hoc in a manner that was not envisaged for the original XYZ format. A human has to read the associated documentation to establish the precise meaning of the annotations, and then write suitable code to extract the annotations to render them useable for e.g. metadata. It is in general uncertain whether software that has been written to process standard XYZ files lacking annotations could successfully cope with this additional content. At best, one might expect the annotations to be simply discarded, since their semantics are not accessible to such a program, only to a human. At worst, it could render the document entirely unreadable by standard software.
- The individual files themselves contain no information about the procedure used to compute the coordinates. In this regard, it would be quite difficult to use these files to reproduce the original calculation; thus the original program inputs are not available, nor indeed are the original program outputs from the quantum mechanical calculation. Curating such a collection therefore would require bespoke interpretation by a human, which always tends to be an expensive and error-prone solution.

The Harvard Clean Energy project [44] is another recent deposition based on quantum chemical calculations, with a claimed 2.3 million molecules associated with an even more impressive 150,000,000 DFT calculations. Access to any individual calculation on any specific molecule however is available only *via* a search front-end to the database based on specified search parameters. No metadata is exposed on any molecule or its calculation parameters in any standard form and it is difficult to envisage any type of curation that could be successfully applied to such a collection. We think it unlikely that enabling open curation was a design feature of the system, although we also believe that this should be included in future designs of such collections.

The recently announced CERN OpenData Portal [45] is also included here, since the data described is very different from the chemical information described above, both in terms of the cost of its acquisition, and of its size and granularity. The organisational prefix for the collection is *10.7483* and this reveals (in December 2014) 53 entries. A typical entry [46] itself contains 3211 datafiles totalling 3.4 TB in size. Analysing this data requires very specialised software, which is itself assigned a persistent identifier [47]. The software is distributed as a virtual image and is designed to be used in the form of a virtual machine containing all the tools required to acquire and analyse the data. The equivalent in our own implementation is the virtual JSmol container for the chemical data [48], that is made available indirectly in the web browser document object model (DOM) as an HTML5 canvas, rather than as a virtual instance on a computer. Working outside the virtual containers provided by the CERN data portal is unlikely to be useful, whereas for chemistry the JSmol container could be replaced by other containers such as e.g. Avogadro 2 [49].

## Conclusions

This brief survey of two recently published molecular data collections indicates that each subject domain will benefit from specifically optimising the features of repository collections for its own needs. We believe that in the chemistry domain, it is useful to adopt a molecular granularity and to develop metadata, search and acquisition mechanisms appropriate for this granularity, even at a scale of 2.3 million molecules. We think it less useful to aggregate the molecules into single containers for which metadata about individual molecules is not exposed. It is also essential that the procedures adopted are programmatic, in that all the required information to re-curate the dataset is available for machine processing. If this is so, then there is no reason why the process could not scale well beyond 2.3 million molecules if required.

## Code availability

The MOPAC software, including the latest PM7 parameter set [10] can be obtained and licensed from http://openmopac.net. The DSpace software itself is open source [1]. The SpectraDSpace DIM2DataCite crosswalk is archived [50]. The Javascript routines implementing [36] the functionality described in the results [34] section are available via the repository entries cited in ref 36.
